# Automated SSHHPS Analysis Predicts a Potential Host Protein Target Common to Several Neuroinvasive (+)ssRNA Viruses

**DOI:** 10.3390/v15020542

**Published:** 2023-02-15

**Authors:** Katarina Z. Doctor, Elizabeth Gilmour, Marilyn Recarte, Trinity R. Beatty, Intisar Shifa, Michaela Stangel, Jacob Schwisow, Dagmar H. Leary, Patricia M. Legler

**Affiliations:** 1 Navy Center for Applied Research in AI (NCARAI) Information Technology Division, U.S. Naval Research Laboratory, Washington, DC 20375, USA; 2 Computer Science Department, Oberlin College, Oberlin, OH 44074, USA; 3 Biology Department, Winston-Salem State University, Winston-Salem, NC 27110, USA; 4 Biology Department, Norfolk State University, Norfolk, VA 23504, USA; 5 Chemistry Department, US Naval Academy, Annapolis, MD 21402, USA; 6 Center for Biomolecular Science and Engineering (CBMSE), U.S. Naval Research Laboratory, Washington, DC 20375, USA

**Keywords:** SSHHPS, SARS-CoV-2, neuroinvasive, virus, entry, protease, host–pathogen, symptom prediction, sequence-to-symptom, blood–brain barrier, Wnt, GPR124, ADGRA2

## Abstract

Within the viral genome, short stretches of homologous host pathogen sequences (SSHHPS) span the protease cleavage sites. To identify host proteins that may be cleaved during infection, we searched the human proteome for viral protease cleavage sites (~20 amino acids). We developed a *sequence-to-symptom* tool, automating the search and pairing process. We used the viral protein sequence, PHI-BLAST, and UniProt database for gene ontologies and disease relationships. We applied the tool to nine neuroinvasive viruses: Venezuelan and Eastern Equine encephalitis virus (VEEV, EEEV); severe acute respiratory syndrome (SARS, SARS-CoV-2); Middle East respiratory syndrome (MERS); EV-71; Japanese encephalitis virus (JEV); West Nile (WNV); and Zika (ZIKV). A comparison of the hits identified a protein common to all nine viruses called ADGRA2 (GPR124). ADGRA2 was a predicted hit of the 3CL main protease and papain-like protease (PLpro) of SARS-CoV-2. ADGRA2 is an adhesion G protein-coupled receptor and a key endothelial regulator of brain-specific angiogenesis. It is a Wnt7A/Wnt7B specific coactivator of beta-catenin signaling and is essential for blood–brain barrier (BBB) integrity in central nervous system (CNS) diseases. We show the cleavage of the predicted sequences in MYOM1, VWF by the SARS-CoV-2 PLpro; DNAH8 (dynein) by the MERS PLpro; ADGRA2 by the alphaviral VEEV nsP2 protease; and POT1 by the SARS-CoV-2 and MERS PLpro.

## 1. Introduction

Short stretches of homologous host–pathogen sequences (SSHHPS) can be found within the viral protease cleavage sites sequences [1,2,3]. These host or host-like sequences may have been acquired by ribonucleic acid (RNA) recombination events, as these sequences and their host counterparts appear to be of a common origin [4,5]. Using these short sequences, we can search host and reservoir proteomes to find the sequences from which they may have been acquired. While the RNA sequences of the host proteins differ from species to species, the protein sequences in the viral polyprotein are often similar to those of the host over a short stretch of ~6–8 amino acids [2]. These short sequences flanking the scissile bond are recognized by viral proteases, we hypothesized that host proteins carrying similar sequences may be cleaved during infection. Observations of cleaved host proteins by Group IV (+)ssRNA viral proteases date back to the 1980s [2,3,6,7]. One of the earliest examples of host protein cleavage was that of histone H3 by the foot-and-mouth disease viral protease [7]. Interferon (IFN) stimulates the transcription of hundreds of genes in order to attain an antiviral state within the cell and around it; thus, histone tails and histone modifying enzymes are logical and strategic targets for post-translational silencing by viral proteases.

Coronaviruses (CoV) and picornaviruses are known to undergo both homologous and non-homologous recombination events at relatively high frequencies [8]. Host genome(s) serve as one of the largest sources of foreign genetic material; however, convergent evolution also may have occurred in some cases [5]. In CoV and picornaviruses, recombination hot spots are non-randomly distributed in the RNA genome [9]. Polymerase “pausing” during the synthesis of viral RNA is thought to occur due to stable RNA secondary structures [8]. These pauses may allow templates to dissociate and rebind the polymerase (template switching). In poliovirus, viral RNA recombination events are thought to occur concomitantly with RNA synthesis as the polymerase jumps from one template to another [10]. There also is evidence of a polymerase-independent mechanism of RNA recombination [11]. In humans, RNA recombination is catalyzed by the multicomponent spliceosome, but for viruses the mechanism(s) are still being investigated.

An earlier analysis of viral protease cleavage site sequences suggested that some of the host proteins that were predicted to be cut by (+)ssRNA viral proteases may be related to symptoms and viral pathogenesis [12]; however, a clear trend was difficult to discern from tables and lists of accession numbers. To date, two methods have been reported for finding the counterparts to these short sequences: first, the use of trained neural networks [12,13,14]; and second, the use of sequence similarity or homology developed by our group [1,2,3].

Most host proteins experimentally shown to be cut by viral proteases have been innate immune-response related [2]; however, some of the proteins predicted by the NetCorona neural network [13,14,15,16] and by SSHHPS analysis [1,3] have not previously been assigned a role in innate immunity and these proteins appear to be related to viral pathogenesis. For example, the neural network identified dystrophin for picornaviruses [12], and SSHHPS identified cardiac myosins and myomesin for SARS-CoV-2 [2]. Cellular data and evidence from biopsies have been consistent with these predictions [17,18,19]. NetCorona and SSHHPS utilize different assumptions. Thus the predicted host protein target lists produced by the two approaches differ [1,2,3,12,13,14,15,20].

Stabell et al. [21] also acknowledged relationships between the cleavability of a host’s STING protein and the appearance of pathology in Dengue-2 infected nonhuman primates (NHP). A lack of cleavability trended with a lack of pathology and low titers. Notably, single amino acid substitutions in STING proteins from other species were capable of making these proteins cleavable in vitro. Thus, these single amino acid species-specific differences in the predicted host proteins were important to retain in our analyses, as not all species are infected or affected to the same extent by these viruses (e.g., Eastern Equine encephalitis virus is highly lethal for horses and humans). For drug discovery, lack of pathology in an animal may mean that the intended challenge material is essentially an attenuated live virus vaccine in the animal model.

Kiemer et al. in 2004 predicted SARS 3CL (3-chymotrypsin-like) main protease (Mpro^SARS^) cleavage sites in host proteins using their NetCorona neural network [13]. Among their predicted cleavage sites were cystic fibrosis transmembrane conductance regulator (CFTR), transcription factors (CREB-RP and OCT-1), and components of the ubiquitin pathway. The CFTR hit suggested a possible relationship to SARS viral pathogenesis [13], but had not been proven biochemically or in cells. However, in their earlier analysis of picornaviral proteases [12], their prediction of dystrophin cleavage was confirmed in an ‘uncleavable mouse’ experiment [22]. Badorff et al. [17,23] demonstrated that Coxsackievirus protease 2A could cut dystrophin both in vitro and in cells, and proposed that cleavage of dystrophin initiated a cascade of events that led to dilated cardiomyopathy. This was the first host protein identified by bioinformatic methods that was shown to be cut in cells. A cleavage-resistant dystrophin knock-in mouse was revealed to have a decrease in sarcolemmal disruption and cardiac virus titer following Coxsackievirus infection linking the 2A proteolytic cleavage of dystrophin to the development of cardiomyopathy [22]. More recently, Scott et al. found several hits related to apoptosis and mRNA processing for Mpro^SARS2^ using NetCorona [14], some of which were experimentally confirmed by Miczi et al. [15].

A downside to neural networks is that they require a large amount of training data. Thus, cleavage sites found in avian, murine, human, porcine, and bovine viruses were used to train NetCorona [13,14]. NetCorona therefore is not virus or species specific. It was intended to predict substrates of the main proteases of coronaviruses in general [14]. SSHHPS analysis, on the other hand, is both virus and species specific. For SSHHPS, no training data are required, making it useful for poorly characterized new and emerging viral pathogens, where large amounts of training data are not available. Thus, the lists of predicted host substrates of different viral proteases can be compared for humans or any other species of interest such as a reservoir species.

Seeing that many comparisons can be made among these viruses and hosts, we have automated SSHHPS analysis and have developed a *sequence-to-symptom* tool that is packaged in Python programming language. Here, we applied the *sequence-to-symptom* analysis to several neuroinvasive viruses: SARS/MERS/SARS2; JEV; EV-71; VEEV/EEEV; WNV; and ZIKV.

In our method, we examine host proteins that contain runs of identical residues that may have arisen from recombination event(s); these are rank-ordered based upon similarity to known viral protease cleavage sites in the polyprotein. Given that there are only a few scissile bond sequences recognized by these viral proteases (Table 1), we postulated that there may be a common core set of targets. For example, the 3CL^SARS2^ and 3CL^EV71^ main proteases cleave at Q↓G, Q↓S, and Q↓A [24,25]. The ZIKV serinyl protease and other flaviviral proteases recognize [KR][R]↓[SG]. The papain-like protease (PLpro)^SARS2^ is an endoprotease and deubiquitinase that cuts at L[KRN]GG↓ sequences. The VEEV nsP2 cysteine protease recognizes [QHF][ED]AGA↓[GAY] (Table 1). A comparison of the lists of predicted host targets produced a common hit called ADGRA2 (adhesion G-protein-coupled receptor A2), also known as GPR124 [26]. We discuss the significance of this hit and confirm the scissile bond of this and other hits that were predicted by the program.

## 2. Materials and Methods for In Vitro Analysis and Verification

### 2.1. Materials

*E.coli* DH5 alpha were purchased from New England Biolabs (Ipswich, MA, USA). *E.coli* BL21(DE3) pLysS cells, BugBuster protein extraction reagent, and cOmplete™ EDTA-free protease inhibitor cocktail tablets were from Millipore-Sigma (St. Louis, MO, USA). Chelating and Q Sepharose were from Cytivia Inc. (Marlborough, MA, USA).

### 2.2. CFP-YFP Substrate Cloning

Substrates were created using primer sets and the SLIC method [27]. Inserts encoding the predicted cleavage sites were incorporated between the cyan and yellow fluorescent proteins in pet15b plasmids. Approximately 20 amino acids were inserted, with at least six C-terminals to the scissile bond. One exception was MYOM1, as this cleavage site sequence occurred at the C-terminus and only five residues of the C-terminal to the scissile bond were present in this substrate. 

### 2.3. CFP-YFP Substrate Expression and Purification

Plasmids were transformed into *E.coli* BL-21 (DE3) pLysS cells. Luria Bertani media (3 L) were inoculated with an overnight culture (50 mL). Cells were grown to an OD_600_ of ~1.0 at 37 °C and then induced overnight with 0.3 mM IPTG for ~17 h at 17 °C. Cells were pelleted (6000× *g* for 10 min, 4 °C). Cell pellets were lysed in 100 mL of lysis buffer (50 mM Tris pH 7.6, 500 mM NaCl, 30 mg lysozyme and DNAse, 1 protease inhibitor tablet, and 35% BugBuster), sonicated for 30 s 6 times and centrifuged for 30 min at 20,500× *g* at 4 °C. The supernatant was then loaded onto a nickel-charged Chelating Sepharose column equilibrated with buffer A (50 mM Tris pH 7.6, 500 mM NaCl) and washed with 20% buffer B for 2–5 column volumes until the A_280_ returned to baseline. Buffer B contained 50 mM Tris pH 7.6, 500 mM NaCl, 300 mM imidazole. The protein was eluted with 100% buffer B. Fractions containing the fluorescent protein substrates were dialyzed against 50 mM Tris pH 7.6, 250 mM NaCl, and then against 50 mM Tris pH 7.6. The protein was loaded onto a Q-Sepharose column equilibrated with 50 mM Tris pH 7.6 and eluted with a step gradient (0 to 1.0 M NaCl). Substrates were typically eluted in 20–30% of the high salt buffer. The purified fractions were inspected on SDS-PAGE gels for purity and MW.

### 2.4. In Vitro Protease Assays

The proteases and substrates were purified as described [1,3,28]. Assays were performed in 50 mM HEPES pH 7.4 and 150 mM NaCl. CoV substrates (2 µM final concentration) were mixed with protease (2 µM final concentration) in the presence or absence of zinc acetate (67 mM final concentration). Reactions were run at R.T. (23 ± 5 °C) for 17 h to 4 days. A C112A MERS PLpro mutant enzyme was used as a control. Alphaviral substrates (10 µM) were mixed with 5 µM protease.

### 2.5. Mass Spectrometry

Tandem mass spectrometry (MS/MS) was obtained by high resolution Orbitrap MS analyzer (ThermoFisher Scientific, Inc., Waltham, MA, USA). Gel bands were cut and digested in gel by AspN, LysC, or arginyl endopeptidase (ArgC) overnight. Peptides were extracted with 0.1% formic acid in 50% acetonitrile and 100% acetonitrile from gel pieces, and dried on the speed vac. Prior to analyzing by LC-MS/MS, peptides were reconstituted in 0.1% formic acid in water (20 µL) and placed in a vial. Subsequently, 3 µL of the peptide mixture was injected into U3000 HPLC coupled to Thermo Scientific Orbitrap Fusion Lumos (ThermoFisher Scientific, Inc., Waltham, MA, USA) equipped with a Nanospray Flex Ion Source and analyzed by shot gun proteomics.

## 3. Automation Process of the SSHHPS Analysis: The *Sequence-To-Symptom* Tool

**Figure 1:**
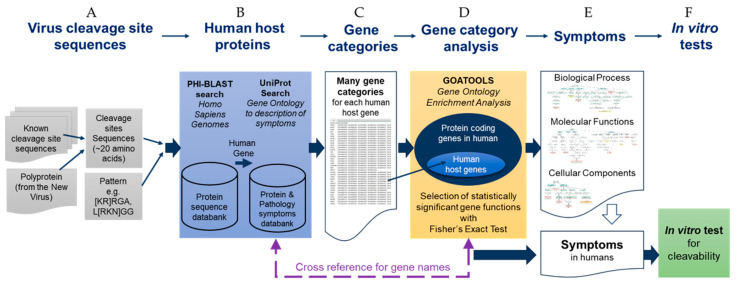
Schematic summary diagram of the automated SSHHPS analysis *sequence-to-symptom* tool with in vitro testing.

To accelerate the analysis of the viral protease cleavage sites in human proteins, we developed a *sequence-to-symptom* tool, automating the search and pairing process. We used the viral polyprotein sequences, PHI-BLAST, the UniProt database for gene ontologies, protein functions, and disease relationships, as well as gene ontology enrichment analysis tools (Figure 1). We packaged all this using Python programming language, and we refer to it as a “*sequence-to-symptom*” tool. The lists that were generated were grouped in Venn diagrams and are available in the Supplementary Materials, Tables S1–S3).

### 3.1. SSHHPS Analysis of Human Proteins

The first step of the sequence-to-symptom algorithm is a PHI-BLAST search using the viral protease clevage site sequences (Figure 1A,B). The input to the sequence-to-symptom algorithm is a ~20 amino acid cleavage site sequence and a pattern (e.g., L[KRN]GG for PLpro^SARS2^) for the PHI-BLAST search. This pattern is common to the viral PLpro cleavage sites of the Group IV (+)ssRNA beta coronaviruses, SARS, MERS, SARS-CoV-2. These PLpro enzymes are also deubiquitinases and deISGylases [29]. The ‘LRGG’ sequence is present in ubiquitin and ISG15 (ISG15 ubiquitin like modifier).

**Figure 2:**
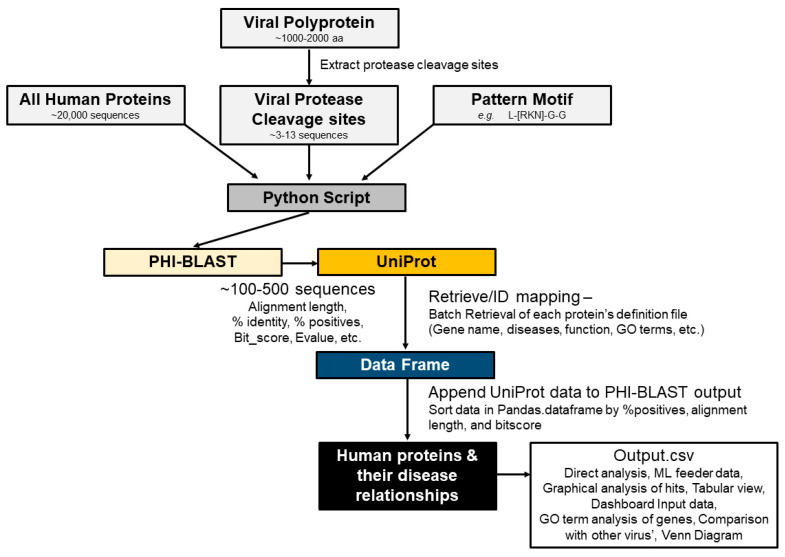
Automation of the SSHHPS analysis.

The results of the PHI-BLAST search are a set of host proteins (i.e., human) with a list of possible cleavage sites. We have appended information from UniProt such as the gene name, function of the protein, related disease states, accession numbers, and gene ontology (GO) terms (Figure 1 and Figure 2). This is followed by sorting by alignment length and percent positives (equivalent to percent similarity) and bitscore.

**Figure 3:**
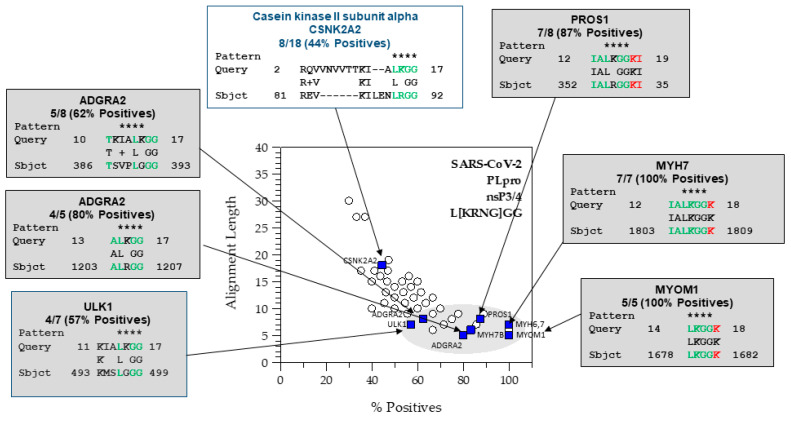
The SARS-CoV-2 PLpro PHI-BLAST hits using a 2D graph of alignment length vs. percent positives. Astrisks show the match to the PHI-BLAST pattern. In green are the residues N-terminal to the scissile bond and in red are the residues C-terminal to the scissile bond that match a viral protease cleavage site sequence. The Python script will generate the most probable host targets based upon subsite tolerances extracted from known cleavage sites (e.g., L[KRNG]GG] for the PLpro^SARS2^). The 2D graph shows a cluster capturing hits with protease cleavage sites and relationships to pathology such as the anti-coagulant protein, PROS1, VWF (overlapped with ADGRA2 at 62.5%, 8), the cardiac myosins (MYH7, MYH6), and sarcomeric protein, MYOM1. The alignments in the upper left are potentially cleavable, but have gaps and shorter runs of matching residues.

The PHI-BLAST search returns several parameters for each protein, such as the expectation value (E-value), bit-score, alignment length, and percent positives. Here, we want to isolate the longest runs of consecutive residue matches around the scissile bond. The E-value describes the number of hits one expects to see by chance from a search of a database of a given size. In this case, a low or high E value did not determine whether a protein was likely to be cleaved. High bit-scores correlate with higher sequence similarity; however, BLAST incorporates gaps to optimize the alignments. Thus, a 2D-graph ofalignment length vs. percent positives can separate the proteins with stretches of similar sequences [2] (Figure 3).

The next step in our sequence-to-symptom algorithm is to search the UniProt database for each of the proteins returned by the PHI-BLAST search. UniProt provides additional information about each protein including the involvement in disease, a description, and related gene ontology terms. The algorithm outputs the UniProt entry title, accession number, alignment length, bitscore, expect value, gaps, number of identities, sequence region that matches, percent positives, query_start (residue number), query_end, subject_start, subject_end, protein_name, function, involvement in disease (Pathology/Biotech), long_protein_name, gene_ontology, and gene_process. We also discarded any incomplete entries (lacking GeneID, UniProt entry, or other necessary information).

It should be noted that BLAST results can vary from run-to-run because most sequence databases are updated daily. Settings for a short sequence search were used (word size = 2; matrix = PAM30; gapcosts = 9, 1; E-value = 200,000; threshold = 11). Note that parameters such as the database searched, substitution matrix, PHI-BLAST pattern, cleavage site length, and gap costs all can be changed by the user and all can alter the outputted list of hits.

With this *sequence-to-symptom* tool, we received many matching proteins for each cleavage site. Sorting by percent positives (sequence similarity), alignment length, and bit-score produced a rank ordered list with the most probable hits moving to the top of the list. Cleavable hits with the highest sequence identity tended to cluster in graphs of alignment length vs. percent positives (Figure 3). We further examined these results using gene ontology enrichment analysis (Figure 1C).

### 3.2. Gene Ontology

Advances in experimental technologies within biostatistics have enabled us to acquire large-scale data. One problem in the biomedical sciences is finding relevant information in public databases, which lack standardization of terms and have a diversity of interfaces and query languages. A widely used ontology at the moment is gene ontology, which develops biological vocabularies. The ontology is applicable to all species, in order to annotate gene products consistently in different databases. Defined gene ontology terms (GO terms) are used for gene annotation [30,31]. Understanding how the ontologies are structured and how functional annotation of gene products is performed is key to understanding their utility. Gene ontologies are derived from descriptions of annotated gene products in different databases.

Gene ontology is a structured and controlled vocabulary for describing biological processes, molecular functions, and cellular components [32]. The GO has approximately 41,000 terms that describe over 4 million genes in almost 470,000 species [31]. The Gene Ontology Annotation project (GOA) is led by the European Bioinformatics Institute (EBI). The creation of GOA has allowed the growth of annotations available in the UniProtKB database.

The GOA project uses manual and automated methods to associate UniProtKB entries with GO terms and provides us access to these annotations. All GO terms have a name and an identifier with the form ‘GO: nnnnnnn’, a description, a process, a definition of the annotation with reference to the source where it was described, synonyms to its description, an ancestor chart, and child term which are descendants of the annotation queried. Some ontology terms are marked as ‘obsolete’. If a term is marked ‘obsolete’, both the term and its identifier are kept in the GO database and there is a comment explaining its expiration and a current non-obsolete term is suggested.

The GO is structured as a directed acyclic graph (DAG) where the nodes are terms, and the edges are relationships between the terms. Structuring the GO as a DAG allows for hierarchical relationships between the GO terms, but as it is not a tree structure, it can show relationships between many different terms. The GO consists of three non-overlapping ontologies: biological process, cellular component, and molecular function. These describe the different roles of the gene. The three ontologies are not connected.

### 3.3. Gene Category Analysis

The UniProt query returns gene categories in the form of GO terms related to the genes (Figure 1C and Figure 2). Most of the genes have many GO terms associated with them, while some have none. These terms are a flat list, rather than a hierarchical structure. A common way to analyze GO terms is through gene ontology enrichment [33,34].

#### 3.3.1. Gene Ontology Enrichment

A gene ontology enrichment analysis is used to test the overrepresentation of gene ontology annotation terms in a list of genes in order to understand their biological significance. Many genomics studies use an enrichment analysis to see if a selected gene set contains enrichment such as over-representation of certain functional classes. The frequency of genes in the sample set is compared to the frequency of the background genes of the species. Different statistical tests can be used to compare the frequency of GO terms; one commonly used test is Fisher’s exact test.

#### 3.3.2. Fisher’s Exact Test

Fisher’s Exact Test is a statistical test to determine if there are non-random associations between two categorical variables. Fisher’s Exact Test is a statistical test used when one has two variables and desires to find out if proportions for one variable are different among values of the other variable. Fisher’s Exact Test of Independence uses a contingency table to display the different outcomes for an experiment. The background gene pool contains the entire list of protein-coding genes of humans.

Fisher’s Exact Test is used to compute uncorrected *p*-values. This *p*-value indicates the probability of seeing at least X number of genes out of a total of N genes in the list annotated to a particular GO term, given the proportion of genes in the whole genome that are annotated with that GO term. That is, the GO terms shared by the genes in the user’s list are compared to the background distribution of annotation. The closer the *p*-value is to zero, the more significant the particular GO term associated with the group of genes is.

$$N=\underset{i}{\sum }R_{i}=\underset{j}{\sum }C_{j}$$

$$p_{cutoff}=\frac{(R_{i}!\;R_{2}!\ldots R_{m}!)\left(C_{1}!C_{2}!\ldots C_{n}!\right)}{N!\;\prod_{i\;j}a_{i\;j}!}$$

A *p_cutoff_* value of ≤0.05 would be deemed significant.

In summary, gene ontology enrichment analysis is used to examine sets of proteins or GO terms to find which are over- or underrepresented compared with a background set. GO enrichment compares the frequency with which GO terms occur in a set compared to the background set. For this study, we use GOATOOLS [35], which is a gene ontology enrichment analysis algorithm in a Python library. GO enrichment returns a set of GO terms (Figure 1D,E). We cross reference the GO terms back to the list of proteins to determine which proteins have overrepresented GO terms associated with them. The terms are segregated into three categories: molecular function (MF), biological process (BP), cellular component (CC). We used our in vitro assay to verify results (Figure 1F).

## 4. Results

### 4.1. Gene Ontology Enrichment–Analysis Results

Our *sequence-to-symptom* code connects the PHI-BLAST results with the function of the protein and its relationship to disease states for *sequence-to-symptom* analysis of viral protease cleavage sites. We included GO terms in our analyses to see if any of the hits were statistically significant.

**Figure 4:**
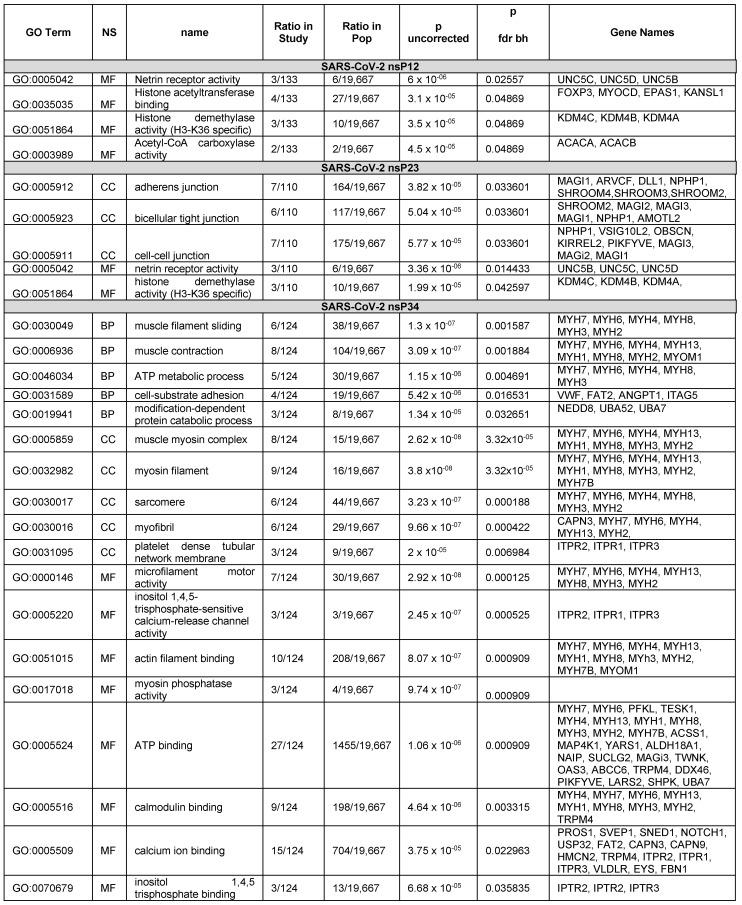
GOATOOL results of the hits from SARS-CoV-2 PLpro nsP1/2 (SGVTRELMRELNGG↓AYTRYV), nsP2/3 (NMMVTNNTFTLKGG*↓APTKVT) and nsP3/4 (VVNVVTTKIALKGG↓KIVNNW) junctional cleavage sites PHI-BLAST searches.

We found GO term enrichment for SARS-CoV-2 PLpro hits related to muscle and cell adhesion, but not blood clotting. The 2D graphical analysis (Figure 3) readily identified proteins related to blood coagulation (VWF and PROS1). VWF and PROS1 were under cell-substrate adhesion and calcium ion binding, respectively (Figure 4). Damage to the myofibrils in cardiomyocytes by SARS-CoV-2 has been observed in cells and autopsy specimens, and anticoagulation therapy is part of the currently recommended guidelines for the treatment COVID-19 [35,36,37]. Thus, both approaches have advantages to analyzing the hits that may be relevant to virus-induced pathology.

**Figure 5:**
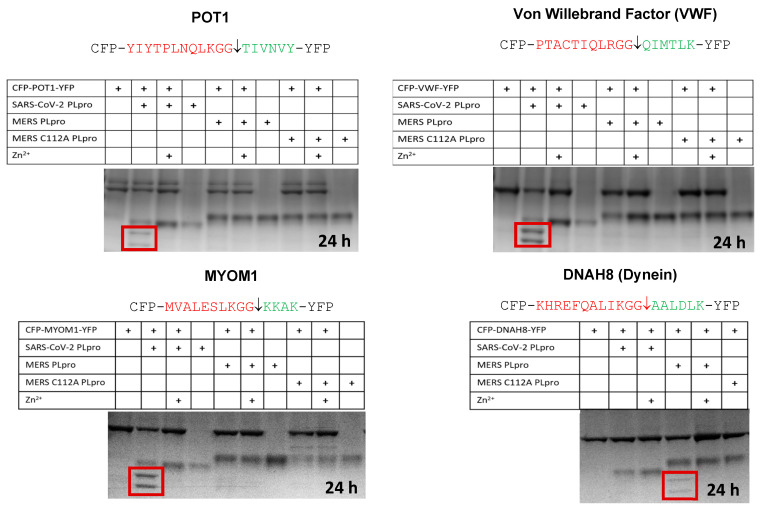
In Vitro cleavage assays using cyan and yellow fluorescent protein substrates for POT1, VWF, MYOM1, and DHAH8 with the SARS-CoV-2 PLpro or the MERS PLpro. Cleavage products are boxed in red. Zinc inhibits the PLpro. The MERS C112A PLpro was used as a control.

We also tested hits that were predicted for MERS but not SARS, and the converse. MYOM1 was predicted to be cut by the SARS-CoV-2 PLpro, while DNAH8 (dynein) was predicted to be cut by the MERS PLpro. Here we observed cleavage of MYOM1 only by the SARS-CoV-2 PLpro but not the MERS PLpro. We also observed cleavage of DNAH8 only by the MERS PLpro, but not by the SARS-CoV-2 PLpro (Figure 5). Only one protein that we selected for in vitro testing was not cleavable (LTBP4) (Table 2).

While the GO term enrichment analyses gave us good results on our small sample set, the table generated by our *sequence-to-symptom* software allowed easy recognition of symptom-related hits and clustering of these hits in 2D plots (Supplemental Materials, Figure S1). The GO term enrichment increased confidence in hits that were in the middle of the list or closer to the mode of the distribution; however, some UniProt entries were missing relevant GO terms. Additionally, some human phenotypes are uncharacterized, but are known for mice (e.g., embryonic lethal phenotypes). Thus, the MGI database can be a useful cross reference for phenotypes that are not present in the UniProt records for the human proteins.

In alphaviruses and other Group IV viruses [38], the order and timing of polyprotein cleavage events affects replication; this is sometimes referred to as an internal clock. The cleavage of each site occurs at different rates [28,39]. Thus, we have segregated the hits by the sites by which they were found and include their relevance to symptoms or pathogenesis.

#### 4.1.1. Analysis of the Hits from the SARS-CoV-2 PLpro nsP1/2 Cleavage Site

Starting with 142 genes, the enrichment analysis process found 12 significant genes in molecular function: ‘FOXP3’, ‘MYOCD’, ‘EPAS1’, ‘UNC5B’, ‘UNC5C’, ‘UNC5D’, ‘ACACA’, ‘ACACB’, ‘KDM4C’, ‘KDM4B’, ‘KDM4A’, ‘KANSL1’. FOXP3 is a transcriptional regulator that is characteristically expressed in T_reg_ cells. T_reg_ cells tamp down inflammation during the resolution phase of an infection. FOXP3 was shown to contain a cleavable sequence in in vitro protease assays [2]. For the nsP1/2 cleavage site, there was an enrichment in proteins that bind histone acetyltransferases and enzymes with histone demethylase activity (H3-K36 specific) and netrin receptor [40] (Figure 4).

#### 4.1.2. Analysis of the Hits from the SARS-CoV-2 PLpro nsP2/3 Cleavage Site

The BLAST query for the nsP2/3 cleavage site returned 179 proteins with potential cleavage sites. The enrichment analysis returned five GO terms with P values less than the cutoff. After referring back to the list of proteins, 19 proteins were found that were associated with the enriched GO terms. The significant genes from enrichment were ‘SHROOM4’, ‘SHROOM3’, ‘SHROOM2’, ‘MAGI3’, ‘MAGI2’, ‘MAGI1’, ‘ARVCF’, ‘DLL1’, ‘NPHP1’, ‘AMOTL2’, ‘OBSCN’, ‘UNC5B’, ‘UNC5C’, ‘KDM4C’, ‘KDM4B’, ‘UNC5D’, ‘KIRREL2’, ‘KDM4A’, ‘PIKFYVE’. Interestingly, the nsP2/3 site produced proteins that were related to adhesion such as adherens junction genes and tight junction genes. However, the adhesion G protein-coupled receptor A2 (ADGRA2) gene was not among the proteins identified by GOATOOLS. ADGRA2 was present in all three hit lists for the cleavage sites. Inspection of the UniProt entry showed that the GO term for adhesion was absent from the ADGRA2 UniProt entry which may be why it was missed. The word ‘adhesion’ was searchable in the UniProt description which was output by our *sequence-to-symptoms* algorithm.

#### 4.1.3. Analysis of the Hits from the SARS-CoV-2 PLpro nsP3/4 Cleavage Site

Starting with 137 genes, the enrichment process found 48 that were significant. Confirmed hits from the in vitro protease assays that were among these were PROS1, MYH6, MYH7, and MYOM1. Significant genes from enrichment were the following: [‘PROS1’, ‘MYH7’, ‘MYH6’, ‘PFKL’, ‘TESK1’, ‘MYH4’, ‘MYH13’, ‘MYH1’, ‘MYH8’, ‘MYH3’, ‘MYH2’, ‘MYH7B’, ‘VWF’, ‘ACSS1’, ‘MAP4K1’, ‘SVEP1’, ‘YARS1’, ‘ALDH18A1’, ‘NAIP’, ‘SNED1’, ‘SUCLG2’, ‘NOTCH1’, ‘USP32’, ‘MAGI3’, ‘FAT2’, ‘ANGPT1’, ‘MYOM1’, ‘CAPN3’, ‘CAPN9’, ‘TWNK’, ‘OAS3’, ‘ITGA5’, ‘NEDD8’, ‘ABCC6’, ‘TRPM4’, ‘DDX46’, ‘ITPR1’, ‘ITPR2’, ‘ITPR3’, ‘VLDLR’, ‘EYS’, ‘UBA52’, ‘PIKFYVE’, ‘LARS2’, ‘SHPK’, ‘UBA7’,’HMCN2’, ‘FBN1’]. The nsP3/4 hits are shown in Figure 4. Many of the hits from the nsP3/4 site were cardiovascular and blood related proteins. GO terms associated with “muscle filament sliding” and “muscle contraction” were in the BP. However, while VWF and PROS1 have relationships to blood clotting disorders and are annotated with the GOterm for blood coagulation (GO:0007596), blood coagulation was not found to be enriched. VWF and PROS1 did have other GOterms that were enriched. In our graphical and tabular sorting methods for SSHHPS analysis; PROS1 was one of our highest predicted substrates (Figure 3), and was at the top of our sorted PHI-BLAST hit lists. Deficiencies in PROS1 in humans lead to a variety of blood clots (e.g., strokes, DVTs, pulmonary embolisms). COVID coagulopathy and thrombosis were associated with worse prognosis in hospitalized patients and anticoagulants reduced mortality by 48% at day 7 [35]. Elevated D-dimer levels were also associated with poor prognosis [35].

We previously showed that the cardiac myosins MYH6 and MYH7 were cleaved in vitro [2]; here, we also found MYOM1 to be cleavable. All are components of the sarcomere. MYOM1 forms the M-band in the sarcomere. The SARS-CoV-2 PLpro may play a role in COVID-related heart damage and heart failure, blood clotting, excessive inflammation, and low blood oxygen levels as the heart plays an important role in the oxygenation of the blood. MYOM1 is not tissue specific and can be found in every skeletal muscle. In contrast, MYOM2 is primarily expressed in cardiac muscle and MYOM3 is mainly expressed in intermediate muscles and specific regions of the cardiac muscle. Perez-Bermejo, et al. showed that the myofibrils in cardiomyocytes were cut into small fragments after SARS-CoV-2 infection [18]. Interestingly, titin and MYOM1 were both predicted for EEEV.

### 4.2. In Vitro Cleavage of POT1, VWF, MYOM1, and DNAH8 by the SARS-CoV-2 or MERS PLpro

To test our results from the *sequence-to-symptom* tool, we constructed substrates containing cleavage site sequences uniquely predicted for the SARS-CoV-2 PLpro and one predicted cleavage sequence for the MERS PLpro. Dynein (DNAH8) was predicted for the MERS PLpro. Both proteases were tested in vitro (Figure 5). The SARS-CoV-2 PLpro cut the C-terminal tail sequence of the sarcomeric protein myomesin-1 (MYOM1) which forms the M-band in myofibrils; it binds myosin, titin, and light meromyosin. Elements of the sarcomere are still distinguishable in SARS-CoV-2 infected cardiomyocytes where the myofibrils are cut into small pieces [18]. In co-stained cardiomyocytes using the thin filament cardiac Troponin T (cTnT) and Z-disk marker α-actinin 2, they observed two bands of cTnT flanking a single α-actinin 2 band [18]. The cTnT troponin doublets may be consistent with MYOM1 and MYH6/MYH7 cleavage, as cleavage of these components would not be expected to disrupt the Z-disc or tropomyosin. The MERS PLpro, as predicted, only cut the DNAH8 sequence. The SARS-CoV-2 PLpro cut the predicted cleavage sites in POT1, VWF, and MYOM1, but not DNAH8. DNAH8 was not predicted for the SARS-CoV-2 PLpro. DNAH8 encodes dynein axonemal heavy chain 8, a protein involved in sperm and respiratory cilia motility.

**Figure 6:**
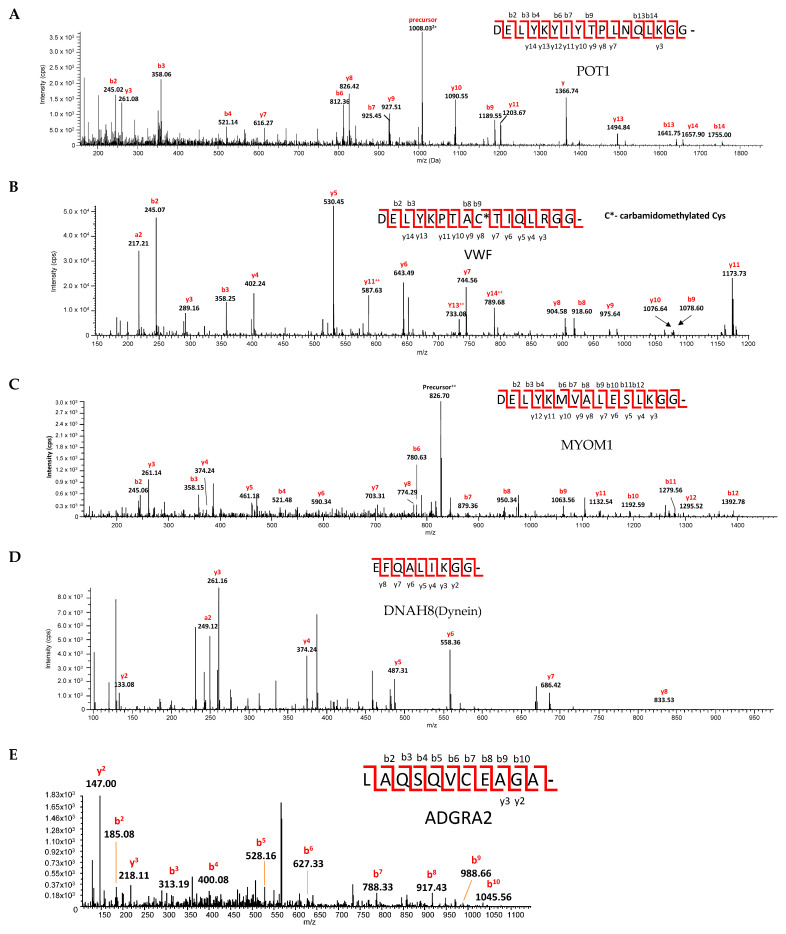
Identification of the scissile bond by tandem LC-MS/MS for SARS-CoV-2 PLpro cut cyan-yellow fluorescent protein substrates containing predicted cleavage sites in (**A**) protection of teleomeres (POT1), (**B**) von Willebrand factor (VWF), (**C**) myomesin-1 (MYOM1), (**D**) dynein axonemal heavy chain 8 (DNAH8), and (**E**) ADGRA2. Cleavage products were separated by SDS-PAGE. All proteins were reduced and alkylated in gel and digested by AspN. Peptide mixtures were analyzed by LC-MS/MS. Spectra of the PLpro cut proteins are shown above. All fragments are singly charged unless they are marked with superscript ++.

The scissile bonds were confirmed by cutting the bands from the SDS-PAGE gels and digesting them with AspN (Figure 6). Each substrate was cut at the predicted site.

### 4.3. Cleavage of an ADGRA2 Sequence by the Alphaviral VEEV nsP2 Protease

**Figure 7:**
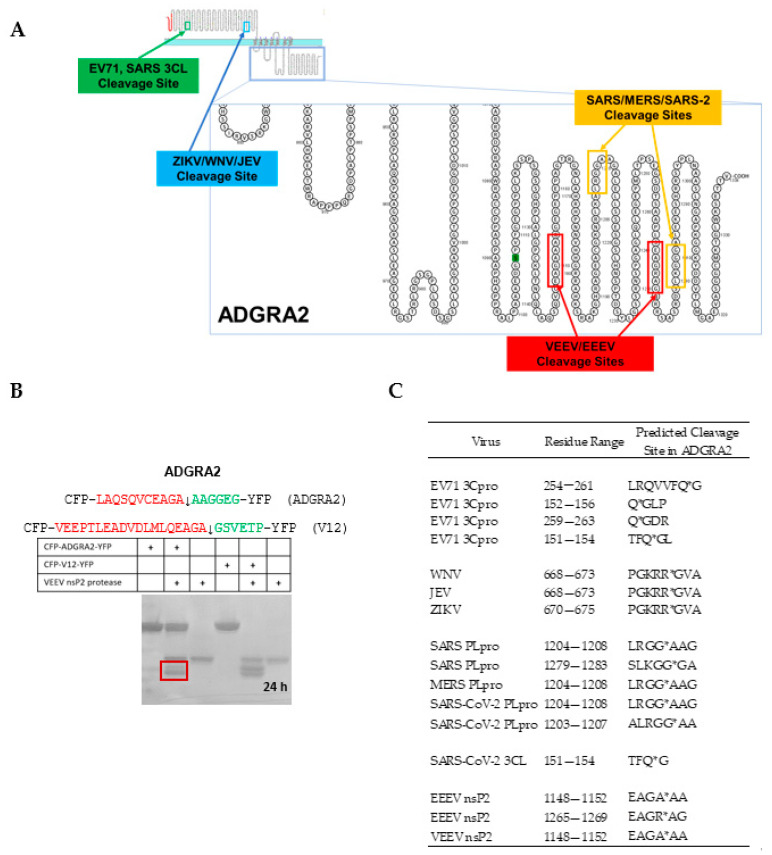
ADGRA2 (GPR124) is a common predicted hit among neuroinvasive viruses. (**A**) ADGRA2 (GPR124) is a G-protein coupled receptor; the regions predicted to be in the cytoplasm by Protter [42] contain many of the predicted cleavage sites. (**B**) A substrate (10 µM) containing one of the two predicted cleavage sites in ADGRA2 for the VEEV nsP2 cysteine protease (5 µM) was cleavable. Cleavage products are boxed in red. The (+) signs indicate which enzymes, inhibitors, or substrates were present in the reaction for the lane in the gel. The V12 substrate (10 µM) contains the natural nsP1/2 cleavage site; this substrate is cut to completion in the 24 h room temperature (23 ± 3 °C) incubation by the VEEV nsP2 cysteine protease (5 µM). Whereas, the cyan-yellow fluorescent protein (CFP-YFP) containing the predicted ADGRA2 cleavage site was more slowly cut during the same incubation period. (**C**) Locations of the predicted viral protease cleavage sites in the ADGRA2 human sequence. Some sites are extracellular, while others are intracellular. Asterisks indicate the scissile bond.

Hit lists of human proteins with predicted cleavage sites were generated with the Python script for a set of neuroinvasive viruses: VEEV, EEEV, SARS, MERS, SARS2, JEV, EV-71, WNV, and ZIKV. Some of the predicted cleavage sites are shown in Figure 7. The cleavage sites were often found in pairs. Two predicted cleavage sites for EV-71 and one site for WNV and SARS-CoV-2 were in the extracellular region, while the other sites were predicted to be in the cytoplasm. Venn diagrams were generated, and a common hit was found among these nine viruses, namely ADGRA2. ADGRA2 is also known as GPR124 and is expressed in endothelial cells. ADGRA2 is an orphan G-protein coupled receptor and was shown to be an essential cofactor along with Reck for Wnt7A/Wnt7b-specific signaling in the mammalian CNS angiogenesis and BBB regulation [26]. Knockout mice show a loss of integrity of the blood–brain barrier under pathological conditions; however, this can be overcome by constitutive activation of Wnt-β-catenin signaling [41], suggesting a potential drug target strategy. Cleavage of ADGRA2 may facilitate or play a role in BBB entry into the brain. For the VEEV nsP2 protease, ADGRB2 was also found among the lists of predicted hits. Cleavage of one of the predicted sites in ADGRA2 was confirmed by the in vitro assay (Figure 7). This substrate was cut more slowly by the VEEV nsP2 cysteine protease than the polyprotein substrate.

### 4.4. Identification of Previously Identified Hits by SSHHPS Analysis

ULK1 and IRF3 were reported to be cut at a LGGG sequence [43,44] by the PLpro^SARS2^ and NEMO at a QV sequence [45,46]. Both proteins contain an LGGG sequence not found in the natural polyprotein sequences. They were therefore not captured by the initial pattern used which was based upon known cleavage sites (L[KRN]GG). Upon addition of ‘G’ to the PHI-BLAST pattern, ULK1 was found in the lists using the nsP3/4 cleavage site. Iterations using broadened patterns may be useful in silico experiments to perform prior to collecting experimental data or for predicting species specific effects.

## 5. Discussion

Proteolytic cleavage of host proteins by viral proteases has been known for almost 40 years. Some cleaved host proteins are present in the nucleus (e.g., histone H3), the cytoplasm, and others have both nuclear import and export sequences (e.g., FOXP3) [47]. The CoV PLpro enzymes are on the endoplasmic reticulum membrane [48]; thus, co-translational cleavage may be possible for CoV. Viral proteases often contain multiple enzyme domains on a single polypeptide chain (e.g., protease, SAM-methyltransferase, helicase are all present in the VEEV nsP2 which is found in both the cytoplasm and nucleus) [1]. The viral proteases associated with host protein cleavages are predominantly from Group IV (+)ssRNA viruses, with the exception of HIV (Group VI, (+)ssRNA retrovirus) [2,3].

The short protease cleavage site sequences consisting of ~6–8 amino acids can be viewed as “host” or “host-like” sequences [1]. Using PHI-BLAST, we can search the human proteome for viral protease cleavage sites. However, ~100s of hits are outputted, even after restricting the hits to those which contain the three to four amino acid PHI-pattern. Thus, sorting was necessary. In our last publication [2], we developed a 2D graphical method to extract the hits with the highest number of similar residues (labeled as % positives in the PHI-BLAST HitList.csv) over the longest alignment length (Figure 3). Cleavable sequences cluster together in the graph. Each point in the graph typically contains more than one protein; the points can be fit to an exponential and a distribution can be extracted. The list of hits could also be sorted by percent positives, alignment length, and bitscore in Excel to identify the host proteins most likely to be cleaved by the viral proteases. The query returns far more proteins than the enzyme will actually cleave, so further work in vitro or in cell-based assays is needed to verify and separate “true” positive results from “false” positive results. Additionally, some of the listed proteins are cell type specific (e.g., cardiac myosins or FOXP3) and may or may not be detected in -omic experiments where a specific cell line is used. The isolation of degradation products in cells is quite challenging; in many cases, the cleavage products are only observable within the first few hours post-transfection or post-infection [1,7]. What is also emerging is that there may be multiple cleavages in various cell types that are relevant to viral pathogenesis and the virus-induced phenotype.

To connect sequence to symptom, we have appended the function and related diseases states (loss-of-function, mutated) to the PHI-BLAST hitlist and have automated the search. Upon inspection of the rank-ordered list and graph, several hits that appear to be related to pathology were found at the top of the list and above the mode of the distribution. The correlation between the host proteins containing viral protease cleavage sites and pathology has been noted by us [1,2,3] and by others [12,13,14,18,21,22,49] and may be useful for biosurveillance *sequence-to-symptom* software for viruses. During the COVID-19 pandemic, millions of SARS-CoV-2 complete and partial sequences were deposited into GenBank; however, only a fraction of the mutations were analyzed and many of the mutations or hotspots within the polyprotein were of unknown significance. The junctional sequences between the nonstructural proteins may hold some key information with regards to viral pathogenesis. The cleavage of human proteins by viral proteases could be viewed as a type of post-translational silencing that transiently results in a loss-of-function and a virus-induced phenotype [1].

We previously showed that the cardiac myosins MYH6 and MYH7 were cleaved in vitro [2]; here, we also found MYOM1 to be cleavable. All are components of the sarcomere. MYOM1 forms the M-band in the sarcomere. The SARS-CoV-2 PLpro may play a role in COVID-related heart damage and heart failure, blood clotting, excessive inflammation, and low blood oxygen levels. The heart plays an important role in the oxygenation of the blood; the cleavage of sarcomere proteins may reduce its ability to pump.

Two methods have been reported for prediction of host protein cleavage sites [2,12,13]; one hit common to both methods was SMG7. SMG7 was predicted for the 3CL^SARS2^ protease [14]. SMG7 is a nonsense mediated mRNA decay (NMD) factor in humans. NMD is a process in which transcripts with premature termination codons are rapidly degraded by an mRNA decay complex. Depletion of NMD components was shown to increase levels of alphaviral proteins and RNA and increase titers of released virus [50].

Notably, the SSHHPS algorithm was able to identify G-PCRs. Approximately 35% of all FDA-approved drugs target G-PCRs. ADGRA2 was found in all nine neuroinvasive viruses, suggesting a possible role in infection. ADGRA2 is an adhesion G protein-coupled receptor and a key endothelial regulator of brain-specific angiogenesis. It is a Wnt7A/Wnt7B specific coactivator of beta-catenin signaling and is essential for BBB integrity [41].

To see if ADGRA2 appeared in hit lists of viruses not strongly associated with encephalitis, we examined the norovirus protease cleavage sites. We did not observe ADGRA2 among the hits; however, ADGRV1 was among the hits for norovirus and the 3CL main protease of SARS-CoV-2 (Supplementary Tables S1–S3). Similarly, for the Old World alphavirus, chikungunya, we also did not find ADGRA2. Only the New World alphaviruses are known to cause encephalitis.

Membrane proteins are often underrepresented in proteomic methods [51]. Thus, the software may be useful in conjunction with proteomic methods and for hypothesis generation to find commonalities among viruses (e.g., maternally transferred, hepatitis, encephalitic, etc.) or novel broad spectrum anti-viral drug targets. The software could be used to remove potential artifacts in proteomic experiments that arise from cellular proteases, amino- and carboxypeptidases. We also found that some of the predicted cleavage sites in ADGRA2 were in the extracellular region (Figure 7). It is unclear as to whether these cleavages would have an activating or inhibitory effect. CoV and alphaviruses have cytopathic effects; thus, we cannot rule out extracellular protease activities that may be relevant to infection and spread.

If in vitro cleavage is observed, the hit may warrant further investigation to determine if the cleavage of the protein is relevant to pathogenesis, infection, or spread. Other experiments beyond the scope of this work, such as the cleavage of full-length protein in virus infected cells in BSL3 containment also may be warranted.

## 6. Conclusions

Here, we have identified a host protein called ADGRA2 (GPR124) with predicted viral protease cleavage sites from a set of nine neuroinvasive viruses, suggesting that the G-PCR or Wnt signaling pathway may be a common target of these Group IV viral proteases. Interestingly, Reck was predicted for Dengue-4, which suggests that other components of the Wnt signaling pathway also may be targeted. ADGRA2 is important for Wnt β-catenin signaling and BBB integrity in CNS disease states [41]. Wnt ligands, antibodies, or small molecules able to activate Wnt signaling may be useful [52] for neuroinvasive viral infections. Interestingly, nature does appear to preserve a common substrate or set of substrates through viral evolution. Lastly, since we only analyzed a small set of neuroinvasive (+)ssRNA viruses, we cannot exclude the possibility that other neuroinvasive viruses may target other GPCRs.

The output files produced by our *sequence-to-symptom* tool could enable follow-on analyses, for example, using machine learning methods or natural language processing to find hidden patterns among viruses that produce similar pathologies or have other similarities in infection. To our knowledge, this is the first *sequence-to-symptom* software for viruses.

## 7. Patents

A patent application has been submitted for the substrates described in this work [53].

## Figures and Tables

**Table 1:** Scissile bonds from several (+)ssRNA viruses.

Family	Scissile Bond Cut by Viral Protease	Examples
*Picornaviridae*	**Q↓G**, Q↓A, Q↓S, Q↓H, Q↓V, Q↓L	Poliovirus, EV68, EV71, Hepatitis A, Foot-and-mouth disease, Coxsackie B virus
*Calciviridae*	**Q↓G**, E↓G, E↓A, Q↓T	Norovirus, Feline calicivirus
*Flaviviridae*	**RR↓G, KR↓G**, RYR↓G, KRR↓(S/T)	JEV (Japanese encephalitisvirus), Dengue, West Nile, Zika (ZIKV), Hepatitis C (HepC), Yellow Fever (YFV)
*Togaviridae*	QE**AGA**↓G, HEAG(CR)↓A, (FY)(DE)**AGA**↓Y, (GA)(GA)↓G	Alphaviruses (VEEV/EEEV/WEEV, Sindbis, SFV, Chikungunya, Ross River), Rubella
*Coronaviridae*	**LKGG**↓, **LRGG**↓, LNGG↓ (PLpro deUb/deISGylase) **Q↓G**, Q↓S, Q↓A, Q↓N (3CL Mpro)	SARS, MERS, SARS-2

In bold are sequences commonly found in the cleavage site sequences.

**Table 2:** Summary of in vitro protease cleavage assay results for SARS-CoV-2 PLpro and VEEV nsP2 protease.

		SARS-CoV-2 PLpro				
Align.		nsp1/2	SGVTRELMRELNGG↓AYTRYV	Cleavage	Host protein target containing	
Length	%Pos	nsp2/3	NMMVTNNTFTLKGG↓APTKVT	In Vitro	a predicted cleavage site	Ref.
(aa)		nsp3/4	VVNVVTTKIALKGG↓KIVNNW			
8	88%		LLIALRGG↓KIEVQL	Y	Vit. K-dep. Protein S (PROS1)	[2]
7	100%		EAEQIALKGG↓KKQLQK	Y	MYH7, cardiac myosin	[2]
7	100%		EAEQIALKGG↓KKQLQK	Y	MYH6, cardiac myosin	[2]
8	88%		PLNQLKGG↓TIVNVY	Y	Protection of telomeres 1 (POT1)	*(this paper)*
7	86%		FQGRDLRGG↓AHASSS	Y	FOXP3	[2]
6	83%		GYCFRELRGG|ECASPL	N	LTBP4	
6	83%		NLTEILNGG↓VYVDQN	Y	ErbB4 (HER4)	[2]
8	63%		ACTIQLRGG↓QIMTLK	Y	VWF	*(this paper)*
5	100%		RMAALESLKGG↓KKAK-	Y	Myomesin-1 (MYOM1)	*(this paper)*
			**MERS PLpro**			
5	80%		KHREFQALIKGG↓AALDLK	Y	DNAH8 (dynein axonemal heavy chain 8)	*(this paper)*
			**VEEV nsP2 Protease**			
Align.		nsP1/2	VEEPTLEADVDLMLQEAGA↓GSVETP	Y		
Length	%Pos	nsP2/3	LSSTLTNIYTGSRLHEAGC↓APSYHV	Y		
(aa)		nsP3/4	TREEFEAFVAQQQRFDAGA↓YIFSSD	Y		
5	80%		LAQSQVCEAGA↓AAGGEG	Y	ADGRA2	*(this paper)*
5	80%		DCFATGRHYWEVDVQEAGA↓GWWVGA	Y	TRIM14	[1]

In red are residues that match a single cleavage site, in green are residues found in other cleavable substrates (e.g., ISG15). Arrow indicates the scissile bond.

## Data Availability

Protease cleavage site prediction hit lists can be found in the Supplementary Materials.

## Supplementary Materials

The following supporting information can be downloaded at https://www.mdpi.com/article/10.3390/v15020542/s1, Tables S1–S3 contain the predicted host targets common to SARS and MERS and hits unique to SARS or MERS, Figure S1 contain graphs of the hits for VEEV, EEEV, and ZIKV, and output files from the software for the viral proteases.

## References

[1] Morazzani E.M., Compton J.R., Leary D.H., Berry A.V., Hu X., Marugan J., Glass P.J., Legler P.M. (2019). Proteolytic cleavage of host proteins by the Group IV viral proteases of Venezuelan equine encephalitis virus and Zika virus. Antivir. Res..

[2] Reynolds N.D., Aceves N.M., Liu J.L., Compton J.R., Leary D.H., Freitas B.T., Pegan S.D., Doctor K.Z., Wu F.Y., Hu X. (2021). The SARS-CoV-2 SSHHPS Recognized by the Papain-like Protease. ACS Infect. Dis..

[3] Hu X., Compton J.R., Legler P.M. (2019). Analysis of Group IV Viral SSHHPS Using In Vitro and In Silico Methods. J. Vis. Exp. JoVE.

[4] Kirkegaard K., Baltimore D. (1986). The mechanism of RNA recombination in poliovirus. Cell.

[5] Gorbalenya A.E. (1992). Host-related sequences in RNA viral genomes. Semin. Virol..

[6] Falk M.M., Grigera P.R., Bergmann I.E., Zibert A., Multhaup G., Beck E. (1990). Foot-and-mouth disease virus protease 3C induces specific proteolytic cleavage of host cell histone H3. J. Virol..

[7] Grigera P.R., Tisminetzky S.G. (1984). Histone H3 modification in BHK cells infected with foot-and-mouth disease virus. Virology.

[8] Lai M.M.C., Holland J.J. (1992). Genetic Recombination in RNA Viruses. Genetic Diversity of RNA Viruses.

[9] Lytras S., Hughes J., Martin D., Swanepoel P., de Klerk A., Lourens R., Kosakovsky Pond S.L., Xia W., Jiang X., Robertson D.L. (2022). Exploring the Natural Origins of SARS-CoV-2 in the Light of Recombination. Genome Biol. Evol..

[10] Copper P.D., Steiner-Pryor A., Scotti P.D., Delong D. (1974). On the nature of poliovirus genetic recombinants. J. Gen. Virol..

[11] Gallei A., Pankraz A., Thiel H.-J.C., Becher P. (2004). RNA recombination in vivo in the absence of viral replication. J. Virol..

[12] Blom N., Hansen J., Blaas D., Brunak S. (1996). Cleavage site analysis in picornaviral polyproteins: Discovering cellular targets by neural networks. Protein Sci..

[13] Kiemer L., Lund O., Brunak S., Blom N. (2004). Coronavirus 3CLpro proteinase cleavage sites: Possible relevance to SARS virus pathology. BMC Bioinform..

[14] Scott B.M., Lacasse V., Blom D.G., Tonner P.D., Blom N.S. (2022). Predicted coronavirus Nsp5 protease cleavage sites in the human proteome. BMC Genom. Data.

[15] Miczi M., Golda M., Kunkli B., Nagy T., Tőzsér J., Mótyán J.A. (2020). Identification of Host Cellular Protein Substrates of SARS-COV-2 Main Protease. Int. J. Mol. Sci..

[16] Zhang S., Wang J., Cheng G. (2021). Protease cleavage of RNF20 facilitates coronavirus replication via stabilization of SREBP1. Proc. Natl. Acad. Sci. USA.

[17] Badorff C., Berkely N., Mehrotra S., Talhouk J.W., Rhoads R.E., Knowlton K.U. (2000). Enteroviral protease 2A directly cleaves dystrophin and is inhibited by a dystrophin-based substrate analogue. J. Biol. Chem..

[18] Perez-Bermejo J.A., Kang S., Rockwood S.J., Simoneau C.R., Joy D.A., Silva A.C., Ramadoss G.N., Flanigan W.R., Fozouni P., Li H. (2008). SARS-CoV-2 infection of human iPSC-derived cardiac cells reflects cytopathic features in hearts of patients with COVID-19. Sci. Transl. Med..

[19] Badorff C., Knowlton K.U. (2004). Dystrophin disruption in enterovirus-induced myocarditis and dilated cardiomyopathy: From bench to bedside. Med. Microbiol. Immunol..

[20] Blom N., Kreegipuu A., Brunak S.r. (1998). PhosphoBase: A database of phosphorylation sites. Nucleic Acids Res..

[21] Stabell A.C., Meyerson N.R., Gullberg R.C., Gilchrist A.R., Webb K.J., Old W.M., Perera R., Sawyer S.L. (2018). Dengue viruses cleave STING in humans but not in nonhuman primates, their presumed natural reservoir. Elife.

[22] Lim B.-K., Peter A.K., Xiong D., Narezkina A., Yung A., Dalton N.D., Hwang K.-K., Yajima T., Chen J., Knowlton K.U. (2013). Inhibition of Coxsackievirus-associated dystrophin cleavage prevents cardiomyopathy. J. Clin. Investig..

[23] Badorff C., Lee G.-H., Lamphear B.J., Martone M.E., Campbell K.P., Rhoads R.E., Knowlton K.U. (1999). Enteroviral protease 2A cleaves dystrophin: Evidence of cytoskeletal disruption in an acquired cardiomyopathy. Nat. Med..

[24] Cui S., Wang J., Fan T., Qin B., Guo L., Lei X., Wang J., Wang M., Jin Q. (2011). Crystal Structure of Human Enterovirus 71 3C Protease. J. Mol. Biol..

[25] Muramatsu T., Takemoto C., Kim Y.-T., Wang H., Nishii W., Terada T., Shirouzu M., Yokoyama S. (2016). SARS-CoV 3CL protease cleaves its C-terminal autoprocessing site by novel subsite cooperativity. Proc. Natl. Acad. Sci. USA.

[26] Cho C., Smallwood P.M., Nathans J. (2017). Reck and Gpr124 Are Essential Receptor Cofactors for Wnt7a/Wnt7b-Specific Signaling in Mammalian CNS Angiogenesis and Blood-Brain Barrier Regulation. Neuron.

[27] Li M.Z., Elledge S.J. (2012). SLIC: A method for sequence- and ligation-independent cloning. Methods Mol. Biol..

[28] Hu X., Compton J.R., Leary D.H., Olson M.A., Lee M.S., Cheung J., Ye W., Ferrer M., Southall N., Jadhav A. (2016). Kinetic, Mutational, and Structural Studies of the Venezuelan Equine Encephalitis Virus Nonstructural Protein 2 Cysteine Protease. Biochemistry.

[29] Freitas B.T., Durie I.A., Murray J., Longo J.E., Miller H.C., Crich D., Hogan R.J., Tripp R.A., Pegan S.D. (2020). Characterization and Noncovalent Inhibition of the Deubiquitinase and deISGylase Activity of SARS-CoV-2 Papain-Like Protease. ACS Infect. Dis..

[30] Rhee S.Y., Wood V., Dolinski K., Draghici S. (2008). Use and misuse of the gene ontology annotations. Nat. Rev. Genet..

[31] Munoz-Torres M., Carbon S., Dessimoz C., Ekunca N. (2017). Get GO! Retrieving GO Data Using AmiGO, QuickGO, API, Files, and Tools. The Gene Ontology Handbook.

[32] Blake J.A., Harris M.A. (2008). The Gene Ontology (GO) project: Structured vocabularies for molecular biology and their application to genome and expression analysis. Curr. Protoc. Bioinform..

[33] Mi H., Muruganujan A., Ebert D., Huang X., Thomas P.D. (2019). PANTHER version 14: More genomes, a new PANTHER GO-slim and improvements in enrichment analysis tools. Nucleic Acids Res..

[34] Klopfenstein D.V., Zhang L., Pedersen B.S., RamC-rez F., Warwick Vesztrocy A., Naldi A.l., Mungall C.J., Yunes J.M., Botvinnik O., Weigel M. (2018). GOATOOLS: A Python library for Gene Ontology analyses. Sci. Rep..

[35] Gomez-Mesa J.E., Galindo-Coral S., Montes M.C., MuC1oz Martin A.s.J. (2021). Thrombosis and Coagulopathy in COVID-19. Curr. Probl. Cardiol..

[36] Martin-Rojas R.M., Perez-Rus G., Delgado-Pinos V.E., Domingo-Gonzalez A., Regalado-Artamendi I., Alba-Urdiales N., Demelo-Rodriguez P., Monsalvo S., Rodriguez-Macias G., Ballesteros M. (2020). COVID-19 coagulopathy: An in-depth analysis of the coagulation system. Eur. J. Haematol..

[37] Panel C.-T.G. Coronavirus Disease 2019 (COVID-19) Treatment Guidelines. National Institutes of Health. https://www.covid19treatmentguidelines.nih.gov/.

[38] Gildenhuys S. (2020). Expanding our understanding of the role polyprotein conformation plays in the coronavirus life cycle. Biochem. J..

[39] Lulla V., Karo-Astover L., Rausalu K., Saul S., Merits A., Lulla A. (2018). Timeliness of Proteolytic Events Is Prerequisite for Efficient Functioning of the Alphaviral Replicase. J. Virol..

[40] Xiao Y., Li B., Zhou Z., Hancock W.W., Zhang H., Greene M.I. (2010). Histone acetyltransferase mediated regulation of FOXP3 acetylation and Treg function. Curr. Opin. Immunol..

[41] Chang J., Mancuso M.R., Maier C., Liang X., Yuki K., Yang L., Kwong J.W., Wang J., Rao V., Vallon M. (2017). Gpr124 is essential for blood-brain barrier integrity in central nervous system disease. Nat. Med..

[42] Omasits U., Ahrens C.H., Muller S., Wollscheid B. (2014). Protter: Interactive protein feature visualization and integration with experimental proteomic data. Bioinformatics.

[43] Mohamud Y., Xue Y.C., Liu H., Ng C.S., Bahreyni A., Jan E., Luo H. (2021). The papain-like protease of coronaviruses cleaves ULK1 to disrupt host autophagy. Biochem. Biophys. Res. Commun..

[44] Moustaqil M., Ollivier E., Chiu H.P., Van Tol S., Rudolffi-Soto P., Stevens C., Bhumkar A., Hunter D.J.B., Freiberg A.N., Jacques D. (2021). SARS-CoV-2 proteases PLpro and 3CLpro cleave IRF3 and critical modulators of inflammatory pathways (NLRP12 and TAB1): Implications for disease presentation across species. Emerg. Microbes Infect..

[45] Hameedi M.A., Prates E.T., Garvin M.R., Mathews I.I., Amos B.K., Demerdash O., Bechthold M., Iyer M., Rahighi S., Kneller D.W. (2022). Structural and functional characterization of NEMO cleavage by SARS-CoV-2 3CLpro. Nat. Commun..

[46] Wenzel J., Lampe J., Müller-Fielitz H., Schuster R., Zille M., Müller K., Krohn M., Körbelin J., Zhang L., Özorhan C. (2021). The SARS-CoV-2 main protease M(pro) causes microvascular brain pathology by cleaving NEMO in brain endothelial cells. Nat. Neurosci..

[47] Magg T., Mannert J., Ellwart J.W., Schmid I., Albert M.H. (2012). Subcellular localization of FOXP3 in human regulatory and nonregulatory T cells. Eur. J. Immunol..

[48] Baez-Santos Y.M., St John S.E., Mesecar A.D. (2015). The SARS-coronavirus papain-like protease: Structure, function and inhibition by designed antiviral compounds. Antivir. Res..

[49] Shiryaev S.A., Ratnikov B.I., Chekanov A.V., Sikora S., Rozanov D.V., Godzik A., Wang J., Smith J.W., Huang Z., Lindberg I. (2006). Cleavage targets and the D-arginine-based inhibitors of the West Nile virus NS3 processing proteinase. Biochem. J..

[50] Balistreri G., Horvath P., Schweingruber C., Zünd D., McInerney G., Merits A., Mühlemann O., Azzalin C., Helenius A. (2014). The host nonsense-mediated mRNA decay pathway restricts Mammalian RNA virus replication. Cell Host Microbe..

[51] Kongpracha P., Wiriyasermkul P., Isozumi N., Moriyama S., Kanai Y., Nagamori S. (2022). Simple But Efficacious Enrichment of Integral Membrane Proteins and Their Interactions for In-Depth Membrane Proteomics. Mol. Cell Proteom..

[52] Martin M., Vermeiren S., Bostaille N., Eubelen M., Spitzer D., Vermeersch M., Profaci C.P., Pozuelo E., Toussay X., Raman-Nair J. (2022). Engineered Wnt ligands enable blood-brain barrier repair in neurological disorders. Science.

[53] Legler P.M., Morazzani E., Glass P.J. (2021). Methods and Compositions for the Detection of Host Protein Cleavage By Group IV Viral Proteases. U.S. Patent.

