# Supplementary material for: Automated SSHHPS Analysis Predicts a Potential Host Protein Target Common to Several Neuroinvasive (+)ssRNA Viruses
Source: Viruses. 2023 Feb 15;15(2):542. doi: 10.3390/v15020542 (PMC9961674; doi:10.3390/v15020542)
Supplement: Supplementary file 1 [file viruses-15-00542-s001.zip › viruses-2119055-supplementary.pdf]

viruses

MDPI

SUPPLEMENTAL INFORMATION

Table S1. Predicted host (human) protein targets common to both SARS-CoV-2 and MERS PLpro enzymes. Highlighted are in vitro confirmed targets.

**POT1**	NAIP	CAPN3	NT5DC3	UBA52	KRT28	AKAP9	KDM4C
KIAA1109	FAM102A	UNC5C	**ADGRA2**	ANKRD31	EPAS1	EXTL1	KDM4B
EFNB3	SUCLG2	HMCN2	TRPM4	PIKFYVE	UNC5B	MEGF10	OSGIN2
CPSF1	PRSS12	UNCX	ITPR2	CD177	CDH22	ACCS	C22orf34
ALDH1A2	CCDC124	ZNFX1	ITPR1	SHPK	UNC5D	RPS19	MTCL1
LAMB3	MAGI3	AMOTL2	PARP1	UBA7	RCL1	PATZ1	KIF12
JPH3	ODAD2	GRIP2	ITPR3	**FOXP3**	INVS	CCT7	NR3C1
RAB23	FAT2	PLAU	SCAP	LTBP4	CARNS1	FSCN2	COL3A1
YARS1	FAM102B	FOCAD	RPL3L	MAGI2	SCUBE2	KCNC3	PPP1R12C
ZC2HC1A	CRY1	VWA5B2	CEP95	MYOCD	GNGT1	SFT2D3	CLEC14A
PRIM2	NCOA2	HJV	ATOH1	CDR2L	NINJ1	LCA10	PKD1L1
KIRREL2	KNL1	TG	ELANE	PUM1	LYPD3	KDM4A	PYCARD-AS1
LOXL3	SUN1	TSTD2	PTPN3	ALK	DNAJB4	CECR2	TRAPPC14
LAYN	PLEKHA5	TMPRSS13					

Table S2. Predicted host (human) protein targets unique to SARS-CoV-2 PLpro. Highlighted are in vitro confirmed targets.

**PROS1**	MYH3	RICTOR	USP32	CAPN9	NEDD8	SLC4A1AP	LRRIQ1	CSTF2
**MYH7**	MYH2	KANSL1	IGHM	LY6L	RUSC1-AS1	BARX2	NRXN3	TCF7L2
**MYH6**	MYH7B	RADX	IGM_HUMAN	ALX3	SLC39A13	DISC1	NT5E	SELENOS
PFKL	**VWF**	ALDH18A1	FGF13	CELF1	KCNMA1	VLDLR	LARS2	
TESK1	ACSS1	LMO7	CFAP206	AKAP13	ABCC6	MDGA2	FBN1	
APBB2	MAP4K1	FREM1	RTKN2	TWNK	ARHGAP32	LRIF1	ALPK3	
MYH4	EHMT2	CEBPZ	ANGPT1	DPP7	TBKBP1	EYS	CRB1	
MYH13	SPOCD1	SNED1	**MYOM1**	FCGBP	VPS13B	VPS13C	TRIP6	
MYH1	FTH1P19	HIRIP3	AMDHD2	CELF2	DDX46	TRAV30	ARHGEF40	
MYH16	SVEP1	NOTCH1	ZCCHC2	OAS3	ICOS	FTHL17	RNF169	
MYH8	CZIB	MN1	TMEM81	ITGA5	GPRC5B	CHTOP	BAHCC1	
ACACB	ACAD8	SP140L	GALNT16	PRDM8	CDK16	TET3	ROR1	
PPARD	CPEB4	ZSWIM8	LRP2	ZFP91	RBPJ	SLC9A3R2	ZBTB39	
RNASE10	CCR10	EIF2S3B	EPHA8	GIMAP2	TAB3	TAPBP	MEPCE	
HHIPL2	RBMX	EIF2S3	TMEM121B	MGAT5B	RAD17	WWTR1	GALNT14	
JMY	EXOC8	KLF16	C9orf106	GALNT2	TESMIN	ACACA	FAM193A	

Table S3. Predicted host (human) protein targets unique to MERS PLpro. Highlighted is the in vitro confirmed target.

UFSP2	TMPRSS6	TMPRSS7	STT3B	PRSS33	CFAP69	PAPLN	RIBC2	SRCRM_HUMAN
GRK3	LOXL2	KLK3	CRYBG2	ARHGAP45	SDF2L1	IL4I1	KLHL31	EFCAB13
PLAT	ARVCF	COL19A1	COLEC10	WEE2	UBB	DNMT3A	GRIA3	TMPRSS12
PDLIM1	ABCA1	CLPP	GRIA2	TMPRSS2	F10	SPEG	PRSS36	LNX1
PLCE1	C5	TMPRSS11E	MAP3K15	PRSS21	GRIK3	C1R	PGGT1B	PRSS42P
COL6A3	AP1G2	TPI1	COX16	GRIK2	KLKB1	C1S	CD5	CFI
SSC4D	SLC7A4	PRRC2B	CD163L1	OXSM	ARSJ	KLK1	OFD1	DUSP15
CUL9	WDR90	ADGRV1	CST3	CD163	GRIA4	MSR1	MAP3K5	DNAH17
OR4D10	PBLD	DNAH9	SMARCA4	GRIK1	MAP3K6	MFSD10	PER3	TMPRSS9
FREM3	MYOM2	STT3A	PLEKHG2	PCLO	HPN	PLEKHA1	CPTP	PKHD1L1
FREM2	SLC5A7	FAT4	CKM	HGFAC	IGSF10	ACR	PER1	ADAM19
CDX2	SOS2	GRIP1	SALL4	DNAH10OS	CSMD2	CAPN8	FLRT3	MPDZ
IGHMBP2	SPATA5L1	IDH3B	CAPN2	SLC16A10	NSD1	MTR	NT5C3A	AHNAK
CD5L	LAMA2	ARNTL	WDR48	ITM2A	DCHS1	RAPGEF6	BCL9L	RABEPK
UBC	VSIG10L2	SLC34A2	HIP1R	HSPH1	PM20D2	RNF213	MRC1	IL16
GRIA1	PTPN13	TTC26	RHOBTB3	SCRIB	NRROS	URGCP	E4F1	SCN1A
PRDX5	SLC38A11	ACAT1	FLRT2	SLCO4A1	NOS1	PAX4	FAM151A	KLHDC1
ZMYND15	CDC25C	FOXD2	RNF220	EIF2D	LOXHD1	WHRN	IVNS1ABP	MAGI1
VPS13A	ATP7A	SPHK2	RELN	FLRT1	SLC23A2	PODXL2	HSPA4L	CFAP54
HSD11B1	LIPM	ALDH3A1	LRRC10B	ADCY5	ABHD8	LNX2	YARS2	WASHC4
PARD3	THBS2	B4GALNT1	**DNAH8**	SLC1A5	TDRP	HYOU1	HSPA4	CLDN8
MOGAT2	ASB8	SLC22A10	KLHL6	CC2D1A	TSNAXIP1	DLG4	DNAJB13	DLG2
SP140	FOXD3	MFSD6L	WDR1	SUGP2	PCDH7	CDC25A	TMEM260	MIPEP
PDE3B	TLX1	MAN1A2	SLC6A8	GRK2	PLXNA3	SYNJ2BP	PDZD11	SHROOM3
C19orf12	DHFR2	NCOA1	DHFR	PDZRN3	ASH1L	SHROOM4	DLG1	SHROOM2
SEL1L3	WNK3	KCNC2	TPCN1	ZBTB32	ARSD	ILVBL	DLG3	PDZD7
ABCA12	SORCS2	XXYLT1	HNRNPK	KLK14	RASA2	KCNB1	IFT20	PRPF18
CNTN3	KLHL14	SOWAHA	COL6A1	PRSS3P2	C2CD3	TMC5	EEFSEC	GALNS
FSTL4	RANGAP1	DDX59	RAG2	PRSS3	TMEM38B	GZMA	ADAM9	SEC61G
ATP6AP1L	POMT2	CSPG4	ATP7B	PRSS1	KCNB2	SMARCE1	OGA	ATG4C
PPP1R8	XRCC3	PKHD1	ACSL3	PRSS2	GPD1	TMPRSS15	MICAL2	PDZRN4
PANK1	ADAMTSL1	ANP32C	TNFRSF19	SLC26A4	RTCA	BCL6B	ZAN	PLCH2
FLI1	TOX	LAMA5	SNTG1	PPEF2	DDX10	HYAL4	HSPG2	MICALL1
PRKAA2	SUCLG1	TRPM1	MTHFD1	PDE8A	VPS13D	PZP	FASN	CPZ
GCK	PRICKLE1	QPRT	DNAH5	SEPHS2	IDE	CHD3	CST5	XPC
EMILIN1	DUOX1	LPIN1	SLC5A8	FLVCR2	TMEM163	A2M	NAA60	SNRPGP15
SNRPG	TLE2	CYP21A2	B3GALT1	NPHP1	RYR2	MINK1	ATP1A3	UBA1
MYO1A	TRIM56	STAB2	HERC2	GNL2	MRPL10	NDST4	SNRNP200	UGDH

FAM83H	SCN2A	CEP295	CLINT1	DCDC1	ASH2L	LINC01599	C18orf21	XIRP2
ANKRD54	ANKRD11	STAG3	VILL	GAS2L3	MERTK	DEF6	GPR143	ATPSCKMT
RPS27A	DNAH7	SLC22A8	CCDC105	PMEL	NXPE3	ATP1A4	MTA1	EYA4
SLC2A10	PATJ	FBH1	NDST1	PROM2	RHBDD3	PHLPP1	EPHB4	IFI6
GPR1	GALNT15	NHLRC2	CSNK2A2	CEMIP	RBM12B	CHERP	PSIP1	FOXRED1
SLC9A7	FN1	HMCN1	MASTL	ZNF831	HECTD4	PRICKLE2	EPHA6	ADAM22
DIEXF	FBXO42	TARBP2	ARFGEF1	RPS6KA6	AP1S3	MSH6	CRYBG3	DERL1
DHRS9	TTN	ZNF106	RAB3GAP1	JAG2	ASB7	NNT	ATP6V0B	F7
OBI1	OXCT1	GINS2	SEMA5A	ZFC3H1	STPG4	AP5M1	GUCY2F	PRKCQ
KCNH3	KLHL22	RGS12	KLF8	B3GALT4	KLK11	CCAR2	CNTN2	PRTFDC1
PTCH1	PAPSS1	PAPSS2	KRT25	NBEA	OR2T2	MTF1	TPT1	FAM241A
ITGB2	DAGLB	BMPER	USP34	SLC37A3	GAL3ST4	SLC9A1	RPS6KA3	HSP90AA5P
DPYSL5	SLC35D2	MAP3K1	LAMA3	TBC1D9B	CRK	KLK8	PRDM14	ARHGEF28
RPS6KA1	TBXT	TLE3	PCDH11X	TLE4	ZNF518A	WAS	PLEKHH2	MUC5AC
OBSCN	UGP2	SLC22A3	BBS2	VWA1	RYR3	ZNF292	FAM163B	PIK3C2B
MICAL3	TBX19	URB1	ITGB4	MAP1S	AOC2	PCDH11Y	TLE1	HHIP
PRKCD	AEBP1	PLXNB1	TNFSF8	RPS6KA5	STRN3	FBXO7	RYR1	TRIM45
RPS6KA2	RPS6KA4	RBM12	FBLN7	TRIM66	TNRC6B	FAM83C	PHKB	TGFBR3
NDST2	INSR	LORF2_HUMAN	SDHB	CCT5	ERVK-9	SGK1	ABCC1	ATP8B3
PIK3C2A	ACSF2	TPT1L_HUMAN						

Supplemental Figure S1. Graphs containing hits for VEEV, EEEV, and ZIKV proteases. Graph of alignment length vs. bit score. Darkest color represents highest alignment length and bit score, lightest color represents the lowest alignment length and bit score. All graphs that have proteins labeled in red represent the protein being common to more than one virus.

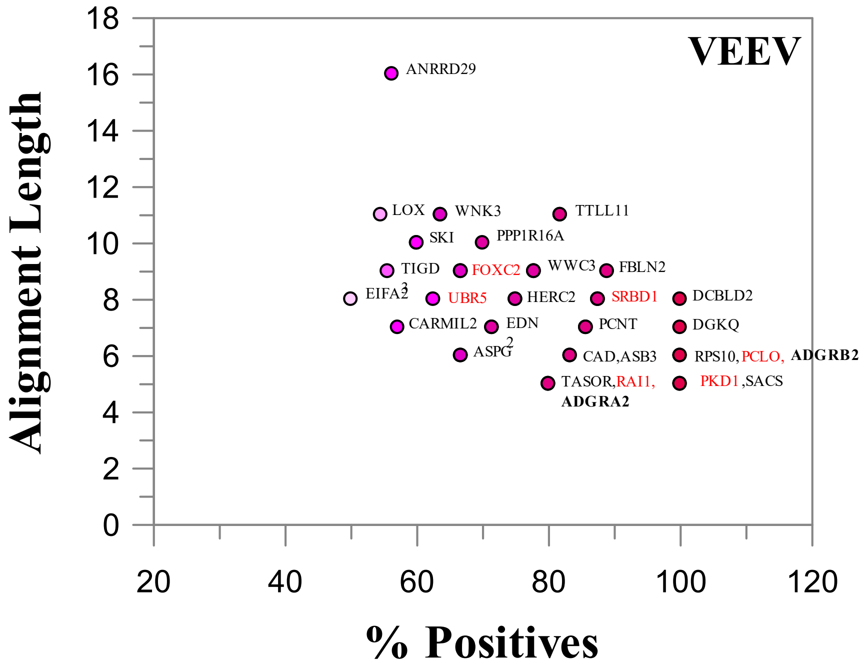

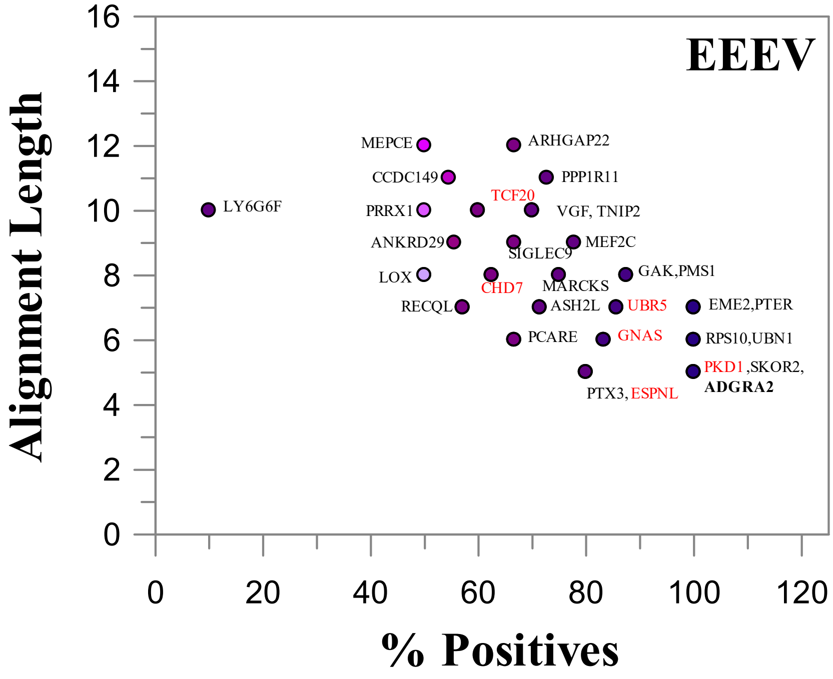
EEEV
Alignment Length
% Positives
0
2
4
6
8
10
12
14
16
0
20
40
60
80
100
120
MEPCE
ARHGAP22
CCDC149
PPP1R11
LY6G6F
TCF20
PRRX1
VGF, TNIP2
ANKRD29
MEF2C
SIGLEC9
LOX
GAK,PMS1
CHD7
MARCKS
RECQL
ASH2L
UBR5
EME2,PTER
PCARE
GNAS
RPS10,UBN1
PKD1 ,SKOR2,
ADGRA2
PTX3, ESPNL

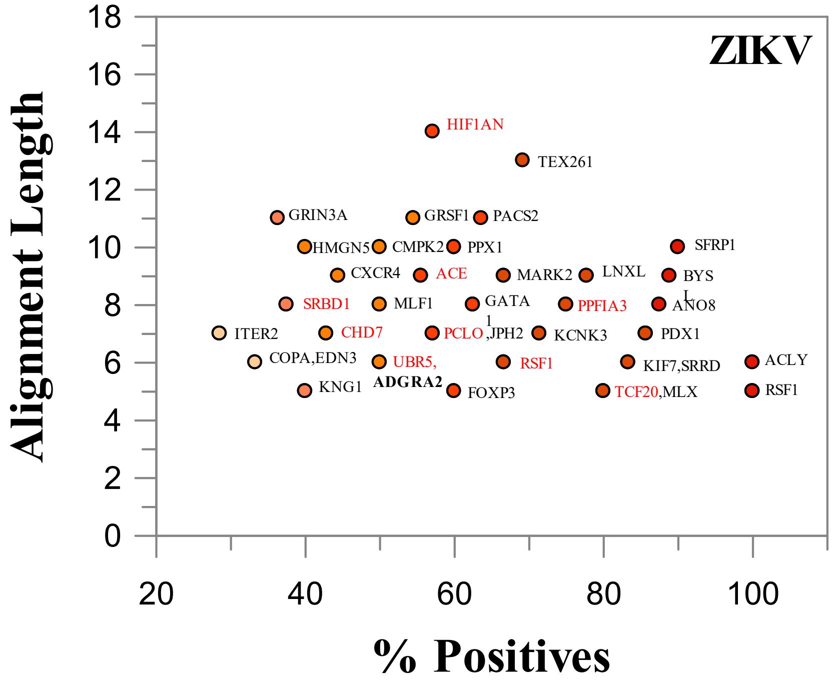
ZIKV
Alignment Length
% Positives
0
2
4
6
8
10
12
14
16
18
20
40
60
80
100
HIF1AN
TEX261
GRIN3A
GRSF1
PACS2
HMGN5
CMPK2
PPX1
SFRP1
CXCR4
ACE
MARK2
LNXL
BYS
SRBD1
MLF1
GATA
PPFIA3
ANO8
ITER2
CHD7
PCLO
JPH2
KCNK3
PDX1
COPA,EDN3
UBR5,
RSF1
KIF7,SRRD
ACLY
KNG1
ADGRA2
FOXP3
TCF20,MLX
RSF1
